# Usefulness of the trabecular bone score in maintenance dialysis patients: A single center observational study

**DOI:** 10.1007/s00508-022-02011-4

**Published:** 2022-03-09

**Authors:** Oliver Malle, Markus Bergthaler, Peter Krisper, Karin Amrein, Hans Peter Dimai, Alexander H. Kirsch, Alexander R. Rosenkranz, Thomas Pieber, Barbara Obermayer-Pietsch, Astrid Fahrleitner-Pammer

**Affiliations:** 1 Department of Internal Medicine, Div. of Endocrinology and Diabetology, Medical University of Graz, Graz, Austria; 2 Department of Internal Medicine, Div. of Nephrology, Medical University of Graz, Graz, Austria

**Keywords:** Osteoporosis, Chronic kidney disease, Mineral and bone disorder, Bone imaging, Bone microarchitecture

## Abstract

**Background:**

The number of dialysis patients is steadily increasing. Associated comorbidities include impaired bone and mineral metabolism, termed chronic kidney disease-mineral and bone disorder (CKD-MBD), leading to a high fracture risk, increased morbidity and mortality and impaired quality of life. While the bone density is assessed with dual-energy X‑ray absorptiometry (DXA), the trabecular bone score (TBS) captures the image texture as a potential index of skeletal microarchitecture. The aim of this study was to evaluate the clinical relevance of DXA and TBS in dialysis patients with and without prevalent fractures.

**Methods:**

Bone disorders were evaluated in 82 dialysis patients (37% female) at the University Hospital of Graz, Austria, by DXA including the assessment of the TBS based on a patient interview and the local routine patient database software. The patient cohort was stratified by having sustained a fragility fracture in the past or not. Descriptive statistics, t‑tests for continuous variables and χ^2^-tests for nominal variables including results of DXA and TBS were performed to compare these groups considering the dialysis modality and duration as well as the number of kidney transplantations.

**Results:**

Of the 82 patients, 32 (39%) had a positive history of fractures. There was a significant association between dialysis duration and fracture prevalence (*p* < 0.05) as well as musculoskeletal pain (*p* < 0.01). No significant correlation between DXA/TBS parameters and musculoskeletal pain could be established. The DXA scores did not correlate with fracture prevalence with the exception of DXA radius measurements; however, fracture prevalence significantly correlated inversely with TBS (*p* < 0.001).

**Conclusion:**

The use of DXA has a limited role in fracture prediction in dialysis patients; however, the TBS seems to add information as an additional tool for fracture risk estimation in this patient population.

## Introduction

Management of bone disorders requires an accurate assessment of a patient’s fracture risk. A major determinant of bone strength is bone mineral density (BMD), which can be assessed with dual-energy X‑ray absorptiometry (DXA) [1]. Only a minority of osteoporosis-related fractures occur in those with a BMD T‑score ≤ −2.5 [2, 3]. Other factors also influence bone strength and fracture risk, including bone geometry, mineralization, turnover and microarchitecture [1, 4, 5].

Additional assessment of bone microarchitecture enhances the accuracy of fracture risk evaluation. Invasive assessment of microarchitectural deterioration is possible through bone biopsy with histomorphometric analysis. As a surrogate noninvasive parameter of microarchitecture, the trabecular bone score (TBS) appears to play an important role in osteoporosis management [6, 7]. The TBS is a noninvasive measurement and easily extracted from DXA lumbar spine images analyzing the variance in gray levels of the DXA image as a function of distance. A unitless value is calculated from the slope of the log-log transform of the 2D variogram, where the slope presents the degree of gray level amplitude variation in the DXA image [8]. A low value represents worse bone trabecular structure and is associated with higher fracture risk [6]. The TBS is an independent predictor of skeletal strength and fragility fracture risk and improves prediction of fracture risk [7]. Advantages of TBS over BMD in the evaluation of bone texture and fracture prediction have been shown in conditions associated with increased fracture risk such as Cushing’s syndrome, corticosteroid therapy, diabetes mellitus, primary hyperparathyroidism, adrenal incidentaloma and anti-aromatase therapy [6, 9, 10]. Thus, considering BMD alone can be misleading in vulnerable populations. For example, in the Manitoba study there were significantly higher BMD values in diabetics than non-diabetics, although there was an increased fracture risk in diabetics [11]. Furthermore, a significantly greater decrease in TBS than BMD has been shown in long-term patients with steroid therapy [12]. A weak association between lumbar spine (LS)-BMD and TBS (e.g. Manitoba study: r = 0.32) suggests that they assess different compartments and aspects of bone quality [13]. The addition of TBS therefore improves fracture risk estimation compared to using each parameter alone [13].

Based on results of the largest meta-analysis currently available (*n* = 17,809) [14] TBS intervention thresholds have been determined. A TBS above 1.31 indicates normal bone structure with low fracture risk, while subjects below 1.23 show degraded bone structure with high fracture risk. Values between 1.31 and 1.23 represent partially degraded bone structure and are classified as intermediate fracture risk [14]. The predictive value of TBS was independent of typical clinical risk factors and BMD [14]. Thus, TBS was incorporated into the World Health Organization fracture risk assessment score (FRAX), resulting in an enhanced estimation of future fracture risk [7].

Chronic kidney disease (CKD) has been shown to have a negative effect on bone microarchitecture leading to a higher fracture risk, with a 2-fold higher fracture risk already at CKD stage 3 [15]. A decrease of kidney function is accompanied by disruption of mineral homeostasis leading to, beside others, abnormalities in phosphorous and calcium concentrations, as well as disruption in parathyroid hormone (PTH) levels and vitamin D metabolism [16]. As a result, severe bone disturbances are almost universally found in end stage kidney disease, termed CKD-MBD [17]. Differences between hemodialysis and peritoneal dialysis have been described, particularly hemodialysis patients show a significantly higher turnover in bone metabolism and a significantly lower BMD, while patients with peritoneal dialysis seem to be less affected by trabecular damage [18].

It remains unclear which method best predicts fracture risk in this population [15]. A bone biopsy with histomorphometric analysis is widely considered as the gold standard diagnostic procedure in CKD-MBD; however, due to its invasiveness and common lack of availability and insufficient expertise, bone biopsies are not recommended routinely in this patient population by guidelines [19]. KDIGO (Kidney Disease: Improving Global Outcomes) 2017 clinical practice guideline suggests BMD testing in patients with CKD. Although low BMD values correlate with future fractures in the general population, BMD may be less useful for risk stratification in CKD patients, as bone microarchitecture changes in CKD are not captured by assessing bone mass alone [17]. Fractures may occur in dialysis patients with normal BMD due to microarchitectural abnormalities. Changes in bone microarchitecture can be evaluated by TBS and its predictive value regarding fractures has already been shown in individuals with CKD [15]. Combining the assessment of both bone mass and bone texture may therefore lead to a more accurate fracture risk estimation in this patient population.

Limited data exist regarding abnormalities in bone structure in CKD patients, particularly regarding effects on the axial skeleton. The current study aimed to compare the clinical relevance of BMD and TBS in dialysis patients regarding associations with prevalent fractures.

## Methods

This study was started in 2014 and approved by the local ethics committee. Inclusion criteria were age between 18 and 90 years, stage 5 CKD, dependence upon dialysis treatment and informed consent. All patients fulfilling these criteria were included. They were recruited from a single tertiary centre (Medical University of Graz, Austria). The exclusion criterion was pregnancy. Anthropometric and clinicopathologic data including age, sex, weight, height, body mass index (BMI), medical and fracture history and current medication were collected through patient interviews using the local routine patient database software as well as the dialysis database and monitor software. All patients were interviewed for fractures in the past including major osteoporotic fractures and peripheral fractures, which included those of the distal femur, tibia, ankle, tibia, ribs and wrist. The assessment of fracture history was based on both the interview and computer database of the patient. Routine X‑rays, e.g. spine X‑rays for detecting asymptomatic vertebral fractures, were not performed. Furthermore, they were interviewed regarding presence of musculoskeletal pain, which was not taken into account, if alternative diagnoses were considered as more likely cause (e.g. osteoarthritis). Fractures, that occurred due to an adequate trauma or prior to diagnosis of CKD, were not taken into account. Dual-energy X‑ray absorptiometry (DXA) was performed in all patients using the GE Lunar iDXA system fan beam bone densitometer (Encore version 16, GE Healthcare, Chicago, IL, USA) at lumbar spine, femur and radius to assess BMD at L1-L4, femoral neck, total proximal femur, proximal 1/3 radius, ultra-distal radius and radius total. Values are presented as grams per centimetre squared (g/cm^2^). In our facility, the least significant change of measurement of bone densitometry is 2.29% for lumbar spine, 3.75% for femur total, and 3.82% for femur neck. Data of young healthy adults from the GE Lunar iDXA system were used as reference group to assess T‑scores based on gender specific peak bone mass. The TBS values were extracted from DXA lumbar spine images using iNsight Software® (version 2.1.2.0; Medimaps, Merignac, France). Biochemical measurements were also performed in all patients assessing PTH, alkaline phosphatase, calcium, ionized calcium, phosphate and 25-OH-vitamin D (using the Liaison assay). The time difference between laboratory tests and DXA was less than 2 months. The patient cohort was divided into two groups: positive fracture history and negative fracture history and descriptive statistics were performed. Data are presented as percentages for categorical variables and mean and standard deviation (SD) for continuous variables. Comparisons between the two groups were performed using independent sample t tests for continuous variables with normal distribution and Mann-Whitney U test with non-normal distribution. A power analysis was performed additionally. To investigate the relation between continuous variables, Pearson’s correlation coefficient was used. The χ^2^-test was used for categorical variables. The statistical software package used was IBM® SPSS® Statistics Version 25 (IBM, Armonk, NY, USA). A *p*-value of less than 0.05 was considered statistically significant.

## Results

The patient cohort consisted of 82 patients, of which 30 (36.6%) were women (Table 1). The mean age was 56.7 ± 14.6 years and the dialysis duration was 73.7 ± 54.3 months. Exactly half of the patient cohort (41 patients) received hemodialysis, the other half peritoneal dialysis and 27 patients (32.9%) previously had kidney transplantation. The most common causes for CKD were glomerulonephritis (27%), diabetic nephropathy (26%), vascular nephropathy (12%) and interstitial nephritis (7%). (Table 2) Except for 3 patients, who had a BMI greater than 40 kg/m^2^, all patients were within the body mass index (BMI) range of 15–37 kg/m^2^ recommended for TBS analysis.

**Table 1:** Baseline characteristics of the patient cohort

Patient characteristics	
*Patients*	82
*Women*	30 (36.6%)
*Men*	52 (63.4%)
*Age, (years)*	56.7 ± 14.6
*Body mass index, kg/m*^*2*^	26.7 ± 5.8
*Dialysis duration, months*	73.7 ± 54.3
*Hemodialysis*	41 (50%)
*Peritoneal dialysis*	41 (50%)
*History of kidney transplantation*	27 (32.9%)
*Musculoskeletal pain*	36 (43.9%)
*Previous fracture*	32 (39.0%)
Proximal femur fracture	5 (6.1%)
Vertebral fracture	14 (17.1%)
Peripheral fracture	19 (23.2%)

**Table 2:** Aetiology of chronic kidney disease in the patient cohort

Etiology of CKD	
Glomerulonephritis	22 (26.8%)
Diabetic nephropathy	21 (25.6%)
Vascular nephropathy	10 (12.2%)
Interstitial nephritis	6 (7.3%)
Postrenal	2 (2.4%)
Amyloidosis	2 (2.4%)
Nephrectomy	2 (2.4%)
Hemolytic uremic syndrome	2 (2.4%)
Hydronephrosis	2 (2.4%)
Pyelonephritis	1 (1.2%)
Focal segmental glomerulosclerosis	1 (1.2%)
Other	11 (13.4%)

Thirty-six patients (44%) patients suffered from musculoskeletal pain. Patients who already underwent kidney transplantation, did not report more musculoskeletal pain (*p* = 0.061) or more fractures (*p* = 0.63). 32 patients (39.0%) had a positive fracture history, of which 5 (15.6%) had a proximal femur fracture, 14 (43.8%) at least one vertebral fracture and 19 (59.4%) any peripheral fracture. Dialysis duration was significant longer in those with positive fracture history (*p* = 0.047) as well as in those who reported musculoskeletal pain (*p* = 0.002), but no association with BMD or TBS values were observed. As expected, all densitometric values as well as TBS significantly correlated with BMI by linear regression (*p* < 0.05). All BMD values as well as TBS did not differ significantly regarding the presence of musculoskeletal pain. No significant associations of calcium metabolism parameters including vitamin D with BMD values or TBS values were observed. There was no significant correlation between laboratory parameter and presence of musculoskeletal pain except ionized calcium (*p* = 0.029) and phosphate (*p* = 0.027). No significant differences were found between patients with hemodialysis and patients with peritoneal dialysis regarding BMD values, TBS or fractures. All BMD values, except femoral neck Z‑score, femur total T‑score and Z‑score, correlated significantly with TBS, and the most significant associations were seen in radius measurements (*p* < 0.001).

In patients with and without prevalent fractures, no significant differences were seen within patient characteristics, medications or laboratory results; however, the presence of musculoskeletal pain was more frequent in patients with prevalent fractures (Table 3). The BMD and TBS values in patients separated by fracture status are listed in Table 4. Regarding previous fractures, there was no significant difference in BMD values except BMD at proximal third of radius and BMD at total radius; however, TBS values differed significantly (*p* < 0.001, statistical power: 95%).

**Table 3:** Baseline characteristics separated by fracture status including fracture type, biochemistry and medication

	Patient characteristics		
	Patients with history of fracture (*n* = 32)	Patients without history of fracture (*n* = 50)	*p*-value
*Women*	13	17	0.54
*Men*	19	33	0.54
*Age, years*	58.6 ± 15.3	55.4 ± 14.2	0.34
*BMI, kg/m*^*2*^	26.1 ± 5.4	27.1 ± 6.1	0.42
*Dialysis duration, months*	84.8 ± 51.7	66.6 ± 55.2	0.14
*Hemodialysis*	20 (62.5%)	21 (42%)	0.07
*Peritoneal dialysis*	12 (37.5%)	29 (58%)	0.07
*History of kidney transplantation*	12 (37.5%)	15 (30%)	0.48
*Musculoskeletal pain*	21 (65.6%)	15 (30%)	**0.002**
**Previous fracture**	32 (100%)	0 (0%)	–
Proximal femur fracture	5 (15.6%)	0 (0%)	–
Vertebral fracture	14 (43.8%)	0 (0%)	–
Peripheral fracture	19 (59.4%)	0 (0%)	–
**Biochemistry**			
Parathyroid hormone, pg/ml	408.3 ± 208.8	407.9 ± 189.2	0.99
Alkaline phosphatase, U/L	89.4 ± 33.8	95.3 ± 41.2	0.48
Calcium, mmol/l	2.24 ± 0.20	2.25 ± 0.18	0.86
Ionized calcium, mmol/l	1.15 ± 0.11	1.15 ± 0.10	0.88
Phosphate, mg/dl	5.16 ± 0.90	5.13 ± 1.22	0.92
25-OH-vitamin D, ng/ml	25.4 ± 12.3	27.3 ± 11.3	0.49
**Medication**			
Cinacalcet	7 (21.9%)	17 (34.0%)	0.24
Phosphate binder: Aluminum salts	2 (6.3%)	2 (4.0%)	0.65
Phosphate binder: Calcium carbonate/acetate	13 (40.6%)	19 (38.0%)	0.81
Phosphate binder: Lanthanum carbonate	7 (21.9%)	9 (18.0%)	0.67
Phosphate binder: Sevelamer hydrochloride/carbonate	8 (25.0%)	20 (40.0%)	0.16

*bold values* reached statistical significance

**Table 4:** Osteodensitometric parameters in the patient cohort separated by fracture status

Osteodensitometry			
	Patients with history of fracture (*n* = 32)	Patients without history of fracture (*n* = 50)	*p*-value
**TBS**	1.081 ± 0.088	1.174 ± 0.119	***p*** **<** **0.001**
**L1-L4**			
BMD, g/cm^2^	0.877 ± 0.140	0.913 ± 0.176	*p* = 0.69
T‑score	−2.0 ± 1.49	−1.86 ± 1.47	*p* = 0.8
Z‑score	−1.36 ± 1.22	−1.16 ± 1.16	*p* = 0.47
**Femur neck**			
BMD, g/cm^2^	0.791 ± 0.127	0.784 ± 0.156	*p* = 0.85
T‑score	−1.96 ± 1.12	−2.06 ± 1.14	*p* = 0.67
Z‑score	−1.13 ± 0.9	−1.32 ± 1.04	*p* = 0.3
**Femur total**			
BMD, g/cm^2^	0.801 ± 0.115	0.809 ± 0.147	*p* = 0.85
T‑score	−1.85 ± 1.15	−2.03 ± 1.16	*p* = 0.42
Z‑score	−1.77 ± 2.47	−1.37 ± 1.12	*p* = 0.71
**Radius ultradistal**			
BMD, g/cm^2^	0.376 ± 0.096	0.402 ± 0.091	*p* = 0.21
T‑score	−3.03 ± 1.81	−2.31 ± 1.74	*p* = 0.08
Z‑score	−2.14 ± 2.03	1.61 ± −1.98	*p* = 0.38
**Radius proximal third**			
BMD, g/cm^2^	0.714 ± 0.141	0.781 ± 0.141	***p*** **=** **0.04**
T‑score	−2.42 ± 1.3	−1.78 ± 1.28	***p*** **=** **0.04**
Z‑score	−1.74 ± 1.11	−1.13 ± 1.31	*p* = 0.05
**Radius total**			
BMD, g/cm^2^	0.537 ± 0.105	0.592 ± 0.117	***p*** **=** **0.04**
T‑score	−2.82 ± 1.5	−2.05 ± 1.54	***p*** **=** **0.04**
Z‑score	−2.06 ± 1.42	−1.42 ± 1.57	*p* = 0.08

bold values reached statistical significance

## Discussion

There are limited data about TBS measurements in CKD patients. This study evaluated the role of TBS in dialysis patients representing a surrogate parameter of bone structural texture. Most patients (68%) showed TBS values < 1.23 indicating a frequent deterioration of bone microarchitecture in this population.

This analysis suggests that adding TBS to BMD improves fracture risk assessment in dialysis populations. A lower TBS was significantly associated with prior fractures, whereas this association was not seen in most BMD values. Only radial BMD values significantly correlated with positive history of fractures but is often not tested in clinical routine. Moreover, the difference of TBS values was even more significant than radial BMD measurements. In addition, the assessment of radial BMD values is often excluded in routine diagnostic. Furthermore, radial BMD measurements have a higher variance compared to measures at hip and spine [16]. Unlike BMD, TBS is also unaffected by overlying calcification, such as vascular calcifications, which is commonly present in dialysis patients [20]. Thus, DXA-derived BMD values alone do not adequately assess the complex microarchitectural abnormalities in dialysis patients and may underestimate future fracture risk in this vulnerable patient population. TBS may provide adequate information of these bone abnormalities not captured by BMD values alone and seems to be an important additional tool when assessing fracture risk. Other imaging modalities, such as QCT, high-resolution peripheral QCT and micro-MRI, also accurately assess microarchitectural deterioration but their use is restricted due to limited availability in clinical practice [16]. TBS requires no additional radiation, is widely available, inexpensive, noninvasive and even available for retrospective analysis of existing DXA lumbar spine images. In our patient cohort, the percentage of patients with T‑score < −2.5 was 40% (lumbar spine), 31% (total hip) and 37% (1/3 radius). In contrast, applying the commonly used TBS threshold of 1.23 [21], an even higher percentage (68%) for high fracture risk was found.

These results are largely in line with already published studies on this topic. Ramalho et al. showed that TBS is an independent predictor of trabecular bone volume and trabecular width and, thus, may adequately represent bone microarchitecture [22]. Dusceac et al. also revealed significantly lower TBS values in dialysis patients matched for BMD indicating deterioration of trabecular microarchitecture [23]. In a retrospective analysis of patients with end-stage kidney disease, lower TBS values were associated with prior non-vertebral fractures [16]. On the contrary, a recent analysis could verify the association of TBS with lower eGFR, but it could not prove an improvement of fracture risk prediction by adding TBS to the diagnostic procedure in patients with eGFR < 60 ml/min/1.73m^2^; however, in patients with eGFR > 60 ml/min/1.73m^2^, it did show a significant positive association between TBS adjusted for FRAX and major osteoporotic fractures [24].

Deteriorations in blood levels of calcium, phosphate, vitamin D and PTH has been shown to be associated with higher risk for cardiovascular events by inducing vascular calcification [25, 26] and to have a catabolic effect resulting in cortical trabecularization as well as high bone turnover and microarchitectural deterioration [27]. Hyperparathyroidism is a CKD-related mineral abnormality and, as expected, common in our patient cohort. Most of the patients (68.3%) showed PTH levels > 300 pg/ml. Interestingly, there was no difference of PTH levels between the two subgroups. This seems quite surprising, as PTH is considered as a substantial surrogate parameter for bone histologic pattern in CKD [28]; however, it should be mentioned that both low and high PTH levels have been shown to be associated with fracture risk [29, 30]. In our study, other biochemical markers that also play a crucial role in bone metabolism, i.e. alkaline phosphatase, calcium, phosphate and 25-OH-vitamin D, also did not differ significantly between the two subgroups.

Our analysis has limitations. As the study cohort of 82 patients was relatively small and from single center, they may represent just a minority of the general dialysis cohort and results may not be easily transferred to the general dialysis population (e.g. peritoneal dialysis 50% vs. 11%; average age 56.7 ± 14.6 years vs. 64.8 ± 14.2 years) [31, 32]. Furthermore, the population was heterogeneous in age, gender and underlying diseases as well as comedication; however, to our knowledge just a few studies exist analyzing the relationship between TBS and clinical, biochemical and radiological parameters in dialysis patients. Considering that fracture history assessment was based just on an patient interview and the local patient data system, particularly asymptomatic vertebral fractures could not be detected and, thus, the fracture prevalence in the non-fracture group could be significantly underestimated. The current study reflects a real-life scenario, but the two subgroups were not matched for age, BMI or gender and therefore comparable only to a limited extent. Of the patients 16 (19.5%) were receiving lanthanum carbonate, which is a known confounder in DXA measurements since it is distributed at the edge of the mineralized bone, but also throughout the mineralized trabecular bone in hyperparathyroid state [33]. This has to be considered when interpreting the results, although the percentage receiving lanthanum carbonate was comparable between the groups. Furthermore, since vertebral fractures are common and asymptomatic among dialysis patients, the missing screening for vertebral fractures at study inclusion represents another limitation.

The two groups may differ regarding other factors not assessed in this analysis contributing to fractures, e.g. frequency of falls, muscle weakness, neuropathy or reduced vision. Furthermore, the accuracy of TBS can be reduced in obese patients due to overlying fat causing textural inhomogeneity [15]. Mean BMI of the patient cohort was 26.7 ± 5.8 kg/m2 and nearly all patients (79 of 82; 96.3%) were within the recommended range for TBS analysis. Given that only three patients were outside the recommended range, we do not assume a relevant impact of precision. Furthermore, the results did not change when these 3 patients were excluded.

This analysis indicates that BMD testing as recommended by the KDIGO 2017 clinical practice guideline may not sufficiently capture the complex microarchitectural deterioration in dialysis patients. TBS, which is influenced by microarchitectural damage, may contribute to the evaluation of bone strength in dialysis patients and enhance fracture risk assessment. Furthermore, we could show a higher significance of TBS compared to BMD further supporting its use in this patient population. These results may encourage further research with prospective studies and larger sample sizes to evaluate the importance of TBS inclusion when performing fracture risk assessment in dialysis patients.

## Conclusion

This study underlines a limited role of DXA in dialysis patients regarding fracture risk assessment as it does not adequately capture the complex microarchitectural deterioration in CKD-MBD. TBS seems to add information as an additional tool in this patient population which is at very high risk for fractures.
